# Social Isolation, Cognitive Function, and Depression Among Chinese Older Adults: Examining Internet Use as a Predictor and a Moderator

**DOI:** 10.3389/fpubh.2022.809713

**Published:** 2022-03-14

**Authors:** Yunjun Li, Xiao Bai, Honglin Chen

**Affiliations:** ^1^Department of Social Work, The Chinese University of Hong Kong, Shatin, China; ^2^Greater Bay Area International Institute for Innovations, Shenzhen University, Shenzhen, China; ^3^Faculty of Social Sciences and Business Studies, University of Eastern Finland, Kuopio, Finland; ^4^Department of Social Work, Fudan University, Shanghai, China

**Keywords:** Internet use, social isolation, older adults, cognitive function, depression

## Abstract

**Objectives:**

Despite the theoretical and practical interest in Internet use among older adults, evidence examining the impacts of Internet use on late-in-life health is limited. This study examines how Internet use affects depression and cognitive function in older adults and investigates if Internet use moderates the relationship between social isolation and depression/cognitive function.

**Method:**

We performed regression analyses using data came from the second wave of the China Longitudinal Aging Social Survey of 2016. Our final sample featured 8,835 older adults.

**Results:**

The results show 11.4% of Chinese older adults often used the Internet to engage in at least one activity. Internet use was negatively associated with depression, but it was positively related to cognitive function. Socially isolated older adults were more likely to have more depressive symptoms and higher level of cognitive function. There was also an interaction effect between Internet use and social isolation on depression/cognitive function. The negative effect of social isolation was stronger for older adults who used the Internet less. The moderating effect of Internet use was significant for both males and females. However, among those who used the Internet more, the depression levels of socially isolated male participants were much lower than female participants.

**Conclusions:**

Our results reveal the importance of considering Internet use in buffering the negative effects of social isolation and the associated health burdens for aging populations. Recommendations for service practice and future research are discussed.

## Introduction

Aging is associated with several stressful life transitions (such as spousal bereavement, retirement, and residential relocation) that lead to an increased risk of shrinking social networks among older adults (1). Such status, defined as social isolation, generally refers to the lack of network size, network diversity, and frequency of contact (2). Accordingly, the unprecedented population aging, coupled with the anticipated loss of intimate relationships and changes in health and social status among older people, suggests that late-life social isolation and associated health problems are emerging as significant public health concerns (3, 4) now exacerbated by the COVID-19 pandemic. Particularly, there is growing concern regarding the effects of social isolation on cognitive function and depression among older adults, in light of healthy aging being pivotal to alleviating burdens of an aging society (5–7). Indeed, numerous studies in Western countries have demonstrated that social isolation puts older people at great risk of depression and cognitive impairment (8, 9); yet studies seldom investigated possible modifying factors in this linkage. Guided by the stress-coping framework, the current study investigated Internet use as a potential moderator and whether it might buffer the negative effects of social isolation on increased depression and cognitive impairment among older adults.

Researchers have proposed the opportunities and benefits the Internet could bring to socially isolated individuals as a promising solution to those challenges, including promoting social connections over remote distances, facilitating online information seeking (e.g., health-related issues), and enhancing competence and autonomy through learning new Internet skills (1, 10, 11). Although previous studies have attempted to identify the association between Internet use and older adults' well-being at an emotional and cognitive level, existing evidence remains equivocal (12, 13). Moreover, most related studies have been conducted in Western countries, with only a few attempts among the Chinese aging population. Besides, a strong recommendation to explore cultural diversity in this topic has already been proposed by researchers, because differences across racial and ethnic groups surrounding Internet use have been documented in the literature (14). For most Chinese older adults, they had entered middle or old age when digital technologies first appeared as a novelty and they began to learn how to use the Internet. Thus, with its late popularization of the Internet amid challenging technical difficulties for older adults, China offers a unique context to advance the global understanding of Internet use.

Given these considerations, including challenges for socially isolated older adults, the potential benefits of Internet use, and the poor computer literacy of Chinese older adults, with a national probability sample of older adults in China, this study aimed to: (a) examine Internet use as a predictor of depression and cognitive function; and (b) investigate Internet use as a moderator in the relationship between social isolation and depression and cognitive function.

### Social Isolation and Internet Use Among Chinese Older Adults

Although Chinese culture has long been recognized as collectivistic or family centered, it has been documented that the traditional family-based model of social networks has gradually weakened for aging parents, partly resulting from the massive internal migration in China (15). According to the China Family Development Report released by the National Health and Family Planning Commission Family Division (16), the “empty-nest elders,” as labeled in the Chinese context and referring to older adults who reside alone or only with their spouse while their children live far away from home (for work or other reasons), account for nearly 50% of older adults, of whom 10% live alone and 41.9% live only with their spouses. Data on the prevalence of social isolation among older adults in the Chinese context remains rare; yet arguably, in view of the “empty nest” phenomenon, socially isolated older adults are very likely to account for a surprisingly high proportion of the population. Moreover, along with the limited socialization after retirement in China and the cultural norm emphasizing interpersonal relationships (17), it is justified to assume that social isolation might provoke more profound adverse effects among Chinese older adults, including higher levels of depression and cognitive impairment, which also makes it urgent to explore corresponding interventions.

As indicated in preceding reviews on the potential benefits of Internet use, older adults in China are increasingly using the Internet with the rapid expansion and the availability of digital technology. In Western countries, Internet use has become more pervasive and integral to the day-to-day functioning of older adults' lives (14, 18). Similarly, in China, an increasing number of older adults have begun to use the Internet. According to the 47^th^ Statistical Report on Internet Development, China had 989 million Internet users as of December 2020, with the percentage of users aged 50 and above rising from 16.9% in March 2020 to 26.3% in December (19). However, not until the 1990s did the Internet and other information technologies rapidly develop in China; Chinese older adults have been called “digital refugees” who generally are unfamiliar with new technologies and less able to use the Internet (17). Thus, unlike their Western counterparts, Chinese older adults' poor computer literacy might provoke more challenging technical difficulties and thereby, increase their negative emotions in the process of Internet use.

### Empirical Evidence on Internet Use and Older Adults' Well-Being

Despite the theoretical and practical interest in Internet use among older adults, researchers have pointed out that the relationship between Internet use and older adults' well-being has not yet been adequately explored and is not well-understood (20).

First, much research has attempted to examine the Internet's association with depression in older adults; yet the findings were mixed (12, 13). For example, in a study of 591 American older adults (50+), Chopik (12) found five Internet activities (i.e., using e-mail, social networking sites, online video/phone calls, online chatting/instant messaging, using a smartphone) were related to fewer depressive symptoms. However, a cross-sectional study exploring the phenomenon of Facebook Depression among 529 individuals aged 18–70+ years demonstrated that older participants were more resilient to the negative effects of Facebook use, especially compared to the younger cohorts (21). Second, despite the strong implications of increased incidence of cognitive decline and disorders in later life and their strong impacts on public health, less is known about the role of Internet use in late-life cognition (22). Also, as Kamin and Lang (23) suggested, there was limited empirical evidence supporting the positive relationship between Internet use and older adults' cognitive function, and most of them were intervention studies with small samples, which calls for more research with a larger sample or longitudinal design to enhance the result's generalizability.

Echoing the aforementioned equivocal findings, many researchers also pointed out the dark side of Internet use has been often overlooked. For example, Ahn and Shin (24) postulated that individuals might spend a substantial amount of time online, even sacrificing time for other valuable activities (e.g., face-to-face communication with family members), thereby reducing meaningful human contact and paradoxically increasing psychological distress. Sum et al. (25) demonstrated that online communication with acquaintances could alleviate older adults' distress, but prolonged Internet use was positively associated with negative mental outcomes. Salovaara et al. (26) highlighted the negative impacts of technical difficulties, that is, it is the process of learning how to use the new and confusing electronic device that might trigger older adults' negative emotions, including anxiety and low self-efficacy. Therefore, one focus of this study was to expand our understanding of the role of Internet use among older adults with a nationally representative sample.

### Stress-Coping Framework and the Moderator Hypothesis

The stress-coping framework has greatly contributed to research on the impacts of stressors on individuals' well-being (27) and therefore, at what level a stressful status like social isolation exerts negative effects would be influenced by older adults' coping resources (namely, a moderator). Theoretically, there is considerable reason for the current study to examine Internet use as a potential moderator in the linkage between social isolation and depression and cognitive function.

As Hofer et al. (10) argued, Internet use could be viewed as a valuable resource for older adults to manage loss, especially for those who face more mobility or activity limitations or frailty. Importantly, it has been reported that social networking was the top usage for Internet users in many countries (21). Scholars have suggested Internet could enable older adults to overcome space limitations, regardless of their frail physical conditions and living locations, thereby allowing them to better connect with the outside world at any time (28). Thus, the Internet could be a tool that increases social support for, improves the social engagement of, and benefits older adults (17, 29, 30). In such cases, it is possible that older adults' pre-existing habits of using the Internet would buffer the effects of social isolation on increasing older adults' depression levels. Meanwhile, according to the information processing model, the Internet has long been recognized as a cultural tool that influences cognitive processes and an environmental stimulus that contributes to the formation of specific cognitive architecture (31). Thus, for socially isolated older adults, it is possible Internet use might mitigate the negative effects of social isolation at the cognitive level. Additionally, though researchers have sought to determine how Internet use affects late-life health outcomes through different pathways (1, 23, 29, 32), few studies have examined such a moderating hypothesis of Internet use. As an exception, in a study of 6,443 community-dwelling older adults (65 or older), Elliot et al. (14) found that the level of technology use moderated the effects of two variables (limitations in ADLs and ill health) on depressive symptoms among participants.

However, considering the aforementioned possible negative impacts of Internet use, there is an imperative need to test the moderator hypothesis of this study; that is, to examine if Internet use acts as a moderator to buffer the negative effects of social isolation on older adults.

### Present Study

Based on the literature review and research gaps described previously, the current study aimed to address two specific research questions using a national probability sample of older adults in China: (1) Is there a relationship between Internet use and depression and cognitive function? (2) Does Internet use moderate the relationship between Internet use and depression and cognitive function?

Moreover, scholars also have suggested that gender differences in the effects of older people's Internet use on their well-being remain underexplored (20, 33), despite evidence that older women and men often use the Internet for different purposes or in different patterns. For instance, older women tend to use the Internet more for communicative purposes, whereas older men are more likely to use the Internet for leisure activities (34). Meanwhile, Chinese studies have also indicated gender as an important predictor of the dependent variables in this study (35, 36). Taken together, the present study further tested the moderating effects based on female and male groups. Thus, from these research purposes and questions, five hypotheses were developed:

H1: Older adults using the Internet more are less likely to have higher depression levels.

H2: Older adults using the Internet more are more likely to have higher levels of cognitive function.

H3: Older adults who are more socially isolated are more likely to have higher depression levels, and Internet use buffers the positive relationship between social isolation and depression.

H4: Older adults who are more socially isolated are less likely to have higher levels of cognitive function, and Internet use strengthens the negative relationship between social isolation and cognitive function.

H5: The moderation model in this study is applicable to both male and female participants.

## Methods

### Data

The China Longitudinal Aging Social Survey (CLASS) is a nationally representative and longitudinal survey of Chinese aged 60 and above. The baseline survey and the recent follow-up survey of the CLASS were fielded in 2014 and 2016, respectively. The 2016 CLASS was the first to include a set of variables measuring Internet use among older adults, so this research used the follow-up data from 2016.

**Figure 1:**
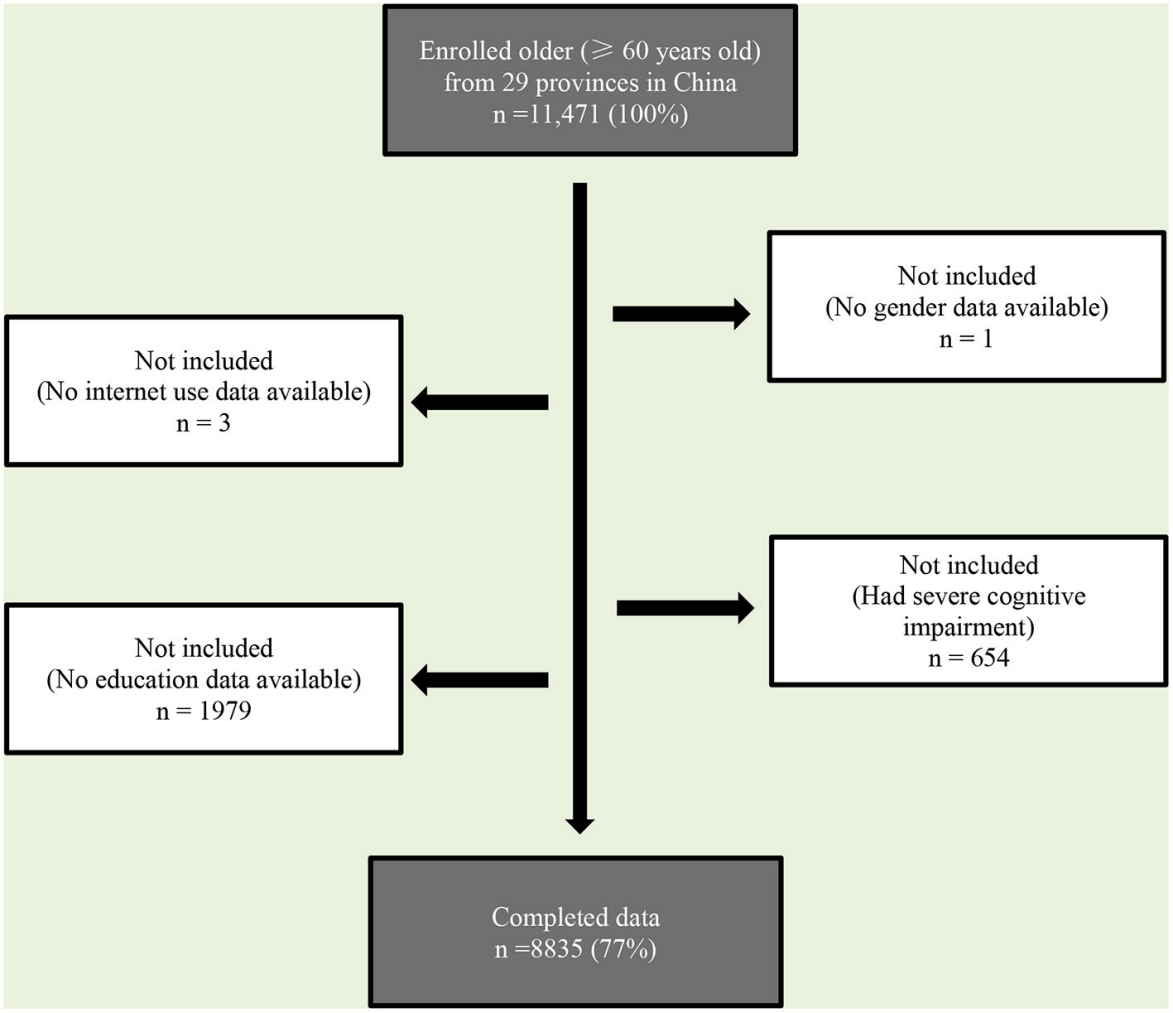
Sample selection flowchart.

The study sample of CLASS was randomly chosen with a three-stage probability proportionate to size sampling method. 134 counties/districts were selected from a sampling frame containing all county-level units in the first stage. In the second stage, 462 villages/communities were drawn at random with the ratio of urban-to-rural population size set at 6:4. In the third stage, older adult per household was randomly selected based on a mapping-and-listing sampling method. The final sample of the baseline survey involved 11,511 respondents. The 2016 CLASS survey successfully tracked 6,603 respondents, with a 57.4% follow-up rate. After supplementing the sample with 4,892 respondents, there were 11,471 sample respondents. After variable screening and data cleaning, the final sample size of this study's moderating model was 8,835 (Figure 1).

### Measurements

#### Internet Use

In the 2016 CLASS questionnaire, participants were asked the following question: “Do you often participate in the activities listed below on the Internet?” The listed items were as follows: “reading current news,” “watching videos,” “chatting with others,” “shopping,” “playing games” and “investing in stocks.” Each answer was either “*no*” (=0) or “*yes*” (=1). This study used the sum scores of these six Internet activities as an indicator of Internet use among older adults. Overall scores ranged from 0 to 6. Higher scores represent higher levels of Internet use. Cronbach's alpha for Internet use was 0.79.

#### Social Isolation

In the CLASS questionnaire, the measure of social isolation was adapted from the Lubben Social Network Scale (37), then validated in Chinese older adults (38). Participants were asked two sets of questions about family and friendship ties, with three items in each set. The questions included: “the number of relatives/friends see or hear from at least once a month,” “the number of relatives/friends they feel able to call for help,” and “the number of relatives/friends they feel at ease to talk about private matters.” Answers were measured on a 6-point Likert-type scale, ranging from 1 (*5 to 8 persons*) to 6 (*no one*). The scores of the six items were summed up and placed on a scale of 6–36. Higher scores indicate higher levels of social isolation. Cronbach's alpha for social isolation was 0.88.

#### Depressive Symptoms

The measure of depressive symptoms was adapted from the Center for Epidemiologic Studies Depression Scale (39), and validated in Chinese older adults (40). Participants were asked about the frequency of depressive symptoms occurring during the past week on a 3-point scale ranging from “*rarely*” (=1), “*sometimes*” (=2), and “*most of the time*” (=3). Sample items included: “Do you feel upset during in the past week?” and “Do you have sleeping problems in the past week?” After three positive items were reverse recorded, the scores of the nine items were summed up and placed on a scale of 9–27. Higher scores indicate higher levels of depression. Cronbach's alpha for depressive symptoms was 0.78.

#### Cognitive Function

The measure of cognitive function was adapted from the abbreviated Short Portable Mental Status Questionnaire (41), and validated for screening for cognitive impairment in Chinese older adults (42). In the CLASS questionnaire, participants' correct answers to listed questions were coded 1, whereas errors were coded 0. Sample items included: “What are the day, month, and year,” “Where are you located now,” and “Can you count backward from 100 by 7's?.” The scores of the eight items were summed up and scored on a scale of 0–16. Higher scores indicate higher levels of cognitive function. Cronbach's alpha for cognitive function was 0.85.

#### Control Variables

Sociodemographic variables, including gender (1 = *female*), age, marital status (1 = *married*), education, ethnicity group (1 = *Han majority*), religious affiliation (1 = *having a religious affiliation*), living status (1 = *living alone*), region (1 = *urban*), personal pension level, number of children, and functional health, were included as control variables. Personal pension level was assessed by a 6-point scale ranging from 1 (*other social endowment insurance*) to 6 (*basic old-age pension system for civil servants*). It corresponded to the schemes of China's pension coverage identified by previous research (43). A higher score indicates a higher level of financial self-sufficiency. This study also assessed functional status using the 6-item activities of daily living (ADL) scale (44) and the 8-item instrumental activities of daily living (IADL) scale (45). Each ADL and IADL item was rated on a 3-point scale ranging from “*on my own*” (=1), “*with help*” (=2), and “*unable*” (=3). Higher final scores indicate higher levels of functional limitations. Cronbach's alphas of ADL and IADL were 0.87 and 0.89, respectively.

### Analysis Plan

Hierarchical regression analysis was applied to investigate Internet use and other related factors in relation to the dependent variables (cognitive function and depression) and to explore the moderating role of Internet use on the association of the independent variable (social isolation) with the dependent variables (46). We estimated four models to predict cognitive function and depression, respectively. In each set of regression analyses, we entered control variables in Step 1 of the model, social isolation in Step 2, Internet use in Step 3, and an interaction term (e.g., social isolation × Internet use) in Step 4. To avoid multicollinearity, we mean centered the moderating variable before creating the interaction term. And, to identify the possible primary and interaction effects of social isolation and Internet use on cognitive function and depression, we examined changes in *R*^2^ from Steps 2–4. The hypothesis of the moderating effect of Internet use would be supported if the interaction was significant (46), and simple slope analysis was conducted to visualize the interaction term. All analyses were conducted in SPSS Statistics 22.

## Results

### Descriptive Statistics

The mean age of the participants was 69.69 years (*SD* = 7.29), with a range of 60 to 106. 51.8% of the participants were male, 72.8 % were married, 23.4 reported illiteracy, 7.3% had an ethnic minority background, and 9.5% had a religious affiliation. The average score for Internet use was 0.25 (*SD* = 0.83), and 7,912 participants (89.6%) reported they didn't often use the Internet for any of these six purposes. Reading current news (9.0%) was the most common activity for older adults to use the Internet, followed by chatting with others (6.9%) and watching videos (4.4%). The prevalence rates of playing games, shopping, and investing in stocks were 2.8, 1.3, and 0.9%, respectively. The socio-demographic characteristics of the participants are summarized in Table 1.

**Table 1:** Descriptive statistics of analytic variables (*N* = 8,835).

	***n* or *M***	**% or *SD***
**Gender**		
Female	4,255	48.2
Male	4,580	51.8
Age (Range: 60–106)	69.69	7.29
**Marital status**		
Married	6,428	72.8
Bereaved, divorced, separated, or never married	2,407	27.2
**Education**		
Illiterate	2,064	23.4
Primary school	250	2.8
Junior school	3,044	34.5
Senior high school	2,140	24.2
Technical school	918	10.4
College or higher	419	4.7
**Ethnicity group**		
Han majority	8,193	92.7
Ethnic minority	642	7.3
**Religious affiliation**		
No affiliation	7,994	90.5
Any affiliation	841	9.5
**Living status**		
Living alone	1,047	11.9
Living with others	7,788	88.1
**Region**		
Rural residence	4,569	51.7
Urban residence	4,266	48.3
Personal pension level (Range: 1–5)	3.28	0.99
Number of children (Range: 0–10)	2.55	1.41
ADL (Range: 11–33)	11.57	1.87
IADL (Range: 8–24)	8.87	2.19
Internet use (Range: 0–6)	0.25	0.83
Reading current news	795	9.0
Watching videos	385	4.4
Chatting with others	607	6.9
Shopping	118	1.3
Playing games	243	2.8
Investing in stocks	82	0.9
Social isolation (Range: 6–36)	21.58	5.75
Cognitive function (Range: 0–16)	13.14	3.26
Depressive symptoms (Range: 9–27)	15.43	3.08

### Moderating Effects

**Figure 2:**
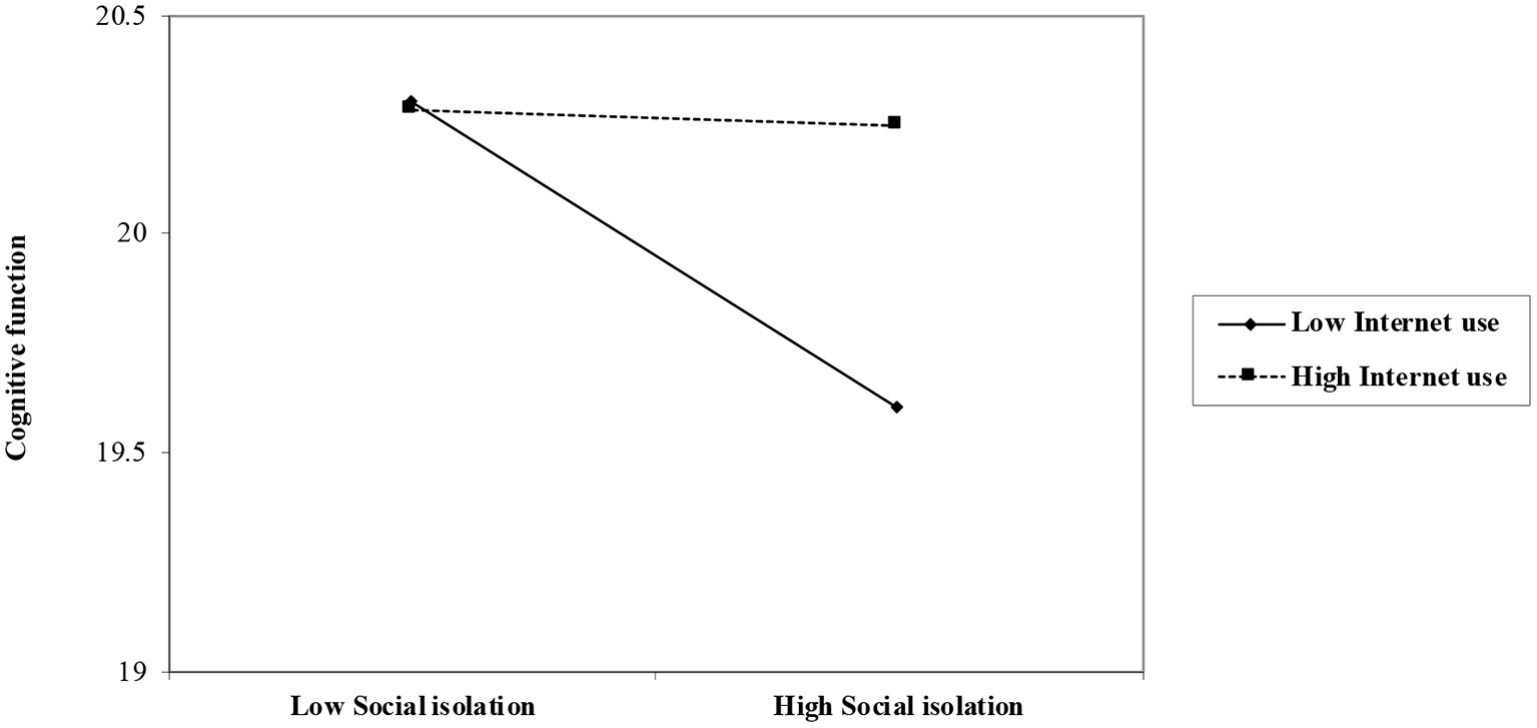
Role of social isolation on cognitive function by levels of internet use among the entire sample (*N* = 8,835).

Table 2 reported the interaction effects of Internet use with social isolation on cognitive function. Model 1 included all sociodemographic variables and depressive symptoms as control variables. The results show older adults who were male, who were younger, who were married, or who were Han majority were more likely to have better cognitive function. Urban residence, education, and personal pension level were positively related to the level of cognitive function while having a religious affiliation, ADL, IADL, and depressive symptoms were negatively related to it. Living status and the number of children were not associated with cognitive function. Model 2 further included social isolation in the model and showed it was negatively associated with cognitive function (β = −0.06, *p* < 0.001). Model 3 included Internet use and demonstrated it was positively related to cognitive function (β = 0.05, *p* < 0.001). Model 4 showed the interaction effect of Internet use and social isolation was significantly positive (β = 0.20, *p* < 0.001). That is, the result indicates a moderating effect of Internet use in the relationship between social isolation and cognitive function, as visualized in Figure 2. The association between social isolation and cognitive function differed according to the level of Internet use. The strength of the negative relationship between social isolation and cognitive function was stronger for older adults who have a lower level of Internet use.

**Table 2:** A hierarchical regression analysis for moderating effects in the relationship between social isolation and cognitive function (*N* = 8,835).

	**Model 1**	**Model 2**	**Model 3**	**Model 4**
	**β**	**β**	**β**	**β**
Female	−0.05^***^	−0.05^***^	−0.05^***^	−0.05^***^
Age	−0.16^***^	−0.16^***^	−0.15^***^	−0.15^***^
Being married	0.04^***^	0.05^***^	0.05^***^	0.05^***^
Education	0.13^***^	0.13^***^	0.12^***^	0.12^***^
Han majority	0.04^***^	0.04^***^	0.04^***^	0.04^***^
Having a religious affiliation	−0.05^***^	−0.05^***^	−0.05^***^	−0.05^***^
Living alone	0.01	0.02	0.02	0.02
Urban residence	0.13^***^	0.13^***^	0.13^***^	0.13^***^
Personal pension level	0.06^***^	0.05^***^	0.05^***^	0.05^***^
Number of children	−0.01	−0.02	−0.01	−0.02
ADL	−0.07^***^	−0.07^***^	−0.07^***^	−0.06^***^
IADL	−0.12^***^	−0.11^***^	−0.11^***^	−0.11^***^
Depressive symptoms	−0.11^***^	−0.11^***^	−0.10^***^	−0.10^***^
Social isolation		−0.06^***^	−0.06^***^	−0.06^***^
Internet use			0.05^***^	−0.14^***^
Social isolation × Internet use				0.20^***^
Adjusted *R*^2^	0.206	0.209	0.211	0.213
Δ*R*^2^		0.003^***^	0.002^***^	0.002^***^

*** *p < 0.001*.

**Figure 3:**
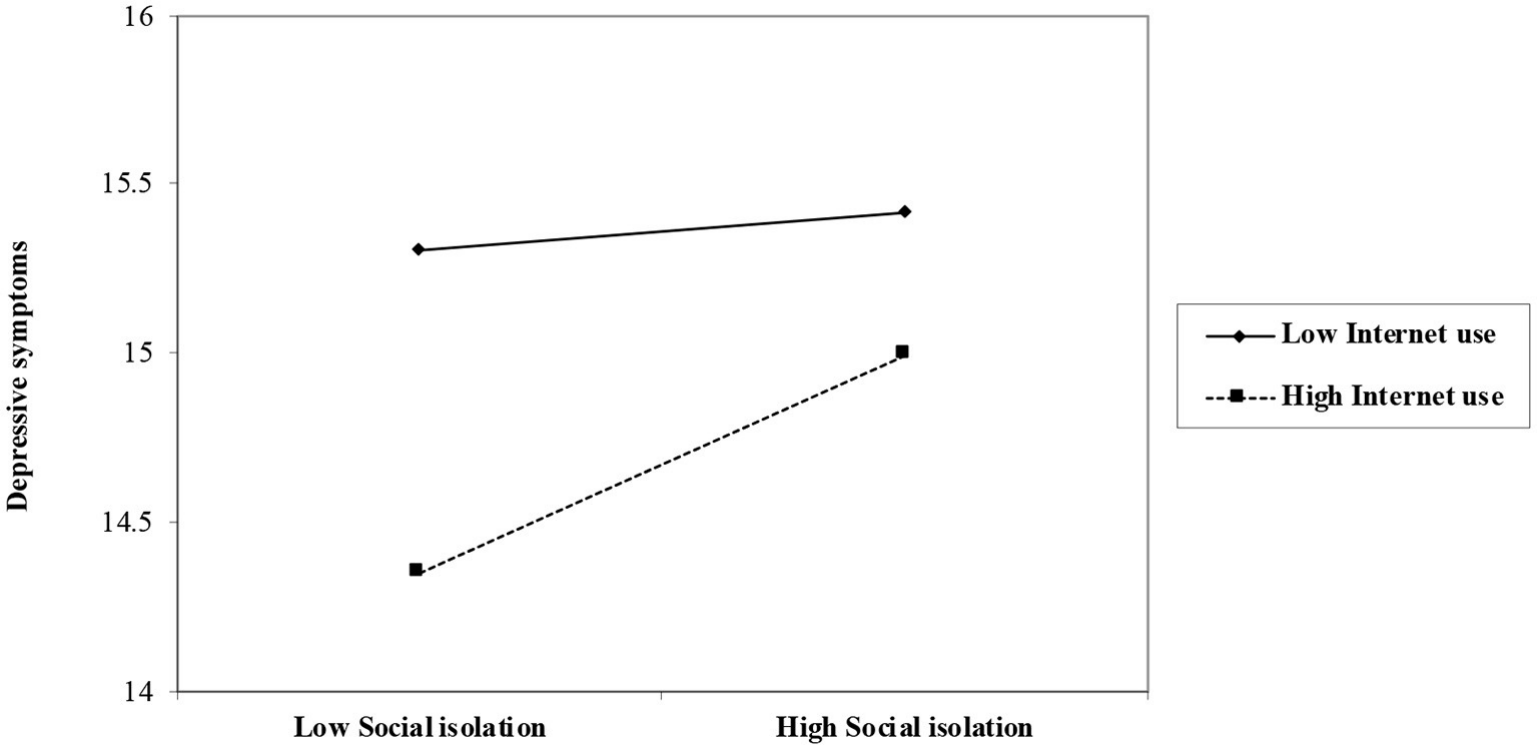
Role of social isolation on depressive symptoms by levels of internet use among the entire sample (*N* = 8,835).

Table 3 provided the results of testing moderating effects of Internet use on the association between social isolation and depression. Similarly, Model 1 included all sociodemographic variables and cognitive function as control variables. The results indicate older adults who were female, who were married, or who were Ethnic minority were less likely to have a high level of depression. Urban residence, education, personal pension level, and cognitive function were negatively related to the level of depression, while living alone status, the number of children, ADL, and IADL were positively related to it. Age and religious affiliation were not associated with depressive symptoms. Model 2's results demonstrate social isolation was positively related to depression (β = 0.06, *p* < 0.001), after controlling for the effects of sociodemographic variables and cognitive function. Model 3 included Internet use and reports it was negatively associated with depression (β = −0.11, *p* < 0.001). Furthermore, Internet use was a more important predictor of depression than social isolation, since the change in *R*^2^ of Step 3 was more substantial than that induced by social isolation in Step 2. Model 4's results indicate the interaction effect of Internet use and social isolation is significantly positive (β = 0.17, *p* < 0.001). As Figure 3 shows, the positive relationship between social isolation and depression was weaker for older adults who have a higher level of Internet use.

**Table 3:** A hierarchical regression analysis for moderating effects in the relationship between social isolation and depressive symptoms (*N* = 8,835).

	**Model 1**	**Model 2**	**Model 3**	**Model 4**
	**β**	**β**	**β**	**β**
Female	−0.03^*^	−0.03	−0.02	−0.02
Age	−0.01	−0.01	−0.02	−0.02
Being married	−0.04^**^	−0.04^**^	−0.04^**^	−0.04^**^
Education	−0.06^***^	−0.06^***^	−0.04^***^	−0.04^***^
Han majority	0.10^***^	0.09^***^	0.09^***^	0.09^***^
Having a religious affiliation	0.01	0.01	0.02	0.02
Living alone	0.08^***^	0.07^***^	0.07^***^	0.08^***^
Urban residence	−0.05^***^	−0.05^***^	−0.04^*^	−0.04^*^
Personal pension level	−0.05^***^	−0.05^***^	−0.04^**^	−0.04^**^
Number of children	0.04^**^	0.05^***^	0.04^**^	0.04^**^
ADL	0.06^***^	0.06^***^	0.07^***^	0.07^***^
IADL	0.08^***^	0.07^***^	0.07^***^	0.07^***^
Cognitive function	−0.13^***^	−0.12^***^	−0.11^***^	−0.12^***^
Social isolation		0.06^***^	0.06^***^	0.06^***^
Internet use			−0.11^***^	−0.27^***^
Social isolation × Internet use				0.17^***^
Adjusted *R*^2^	0.092	0.095	0.105	0.107
Δ*R*^2^		0.003^***^	0.010^***^	0.002^***^

* *p < 0.05,*

** *p < 0.01,*

*** *p < 0.001*.

### Gender-Specific Analyses

**Figure 4:**
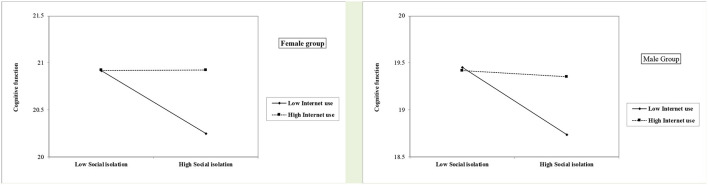
Role of social isolation on cognitive function by levels of internet use among the female and male group.

**Figure 5:**
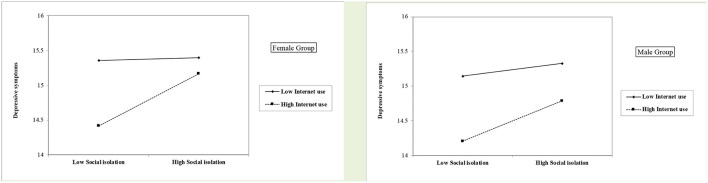
Role of social isolation on depressive symptoms by levels of internet use among the female and male group.

As shown in Table 4, Figures 4 and 5 same as the results of the total-sample analysis, the moderating effects of Internet use on the association between social isolation and cognitive function/depression were significant in both females and males. Notably, as Figure 5 indicates, in the Internet-high-use group, the depression levels of the socially isolated male participants were much lower than for female participants.

**Table 4:** Results of linear regression model for moderating effects in the relationship between social isolation and cognitive function/depression across gender groups (*N* = 8,835).

	**DV: Cognitive function**		**DV: Depression**	
	**Female group**	**Male group**	**Female group**	**Male group**
	**β**	**β**	**β**	**β**
Female	/	/	/	/
Age	−0.18^***^	−0.13^***^	−0.04^*^	0.00
Being married	0.04^*^	0.05^**^	−0.05^**^	−0.02
Education	0.15^***^	0.09^***^	−0.04	−0.05^**^
Han majority	0.03	0.06^***^	0.10^***^	0.09^***^
Having a religious affiliation	−0.04^**^	−0.06^***^	0.01	0.02
Living alone	0.04^*^	−0.01	0.09^***^	0.05^**^
Urban residence	0.15^***^	0.10^***^	−0.03	−0.05^*^
Personal pension level	0.02	0.08^***^	−0.04^*^	−0.03
Number of children	−0.01	−0.02	0.07^***^	0.01
ADL	−0.07^***^	−0.05^**^	0.07^***^	0.06^**^
IADL	−0.09^***^	−0.14^***^	0.07^***^	0.06^**^
Depressive symptoms	−0.10^***^	−0.11^***^	/	/
Cognitive function	/	/	−0.11^***^	−0.13^***^
Internet use	−0.14^*^	−0.15^*^	−0.32^***^	−0.24^***^
Social isolation	−0.05^***^	−0.06^***^	0.06^***^	0.06^***^
Social isolation × Internet use	0.20^***^	0.20^***^	0.23^***^	0.13^*^
Adjusted *R*^2^	0.223	0.195	0.113	0.101

* *p < 0.05,*

** *p < 0.01,*

*** *p < 0.001*.

## Discussion

Although the theoretical and practical interest in Internet use among older adults remains high, the evidence testing its impacts on late-in-life health outcomes is limited (1). Some studies even show it has negative effects on the well-being of older adults (12, 47). This study advances the literature by exploring the interplay of social isolation and Internet use on cognitive function and depression/cognitive function among Chinese older adults.

As our study reports, 11.4% of Chinese older adults often used the Internet to engage in at least one activity (e.g., chat with others). First, the prevalence of Internet use was much lower than rates found in previous studies. For example, Yuan (30) reported a 46.48% use rate among elderly Shanghai residents; however, they lived in the commercial and financial center of mainland China with easy access to computers and smartphones. Second, when compared with Internet use in Western studies–53% in Europe as reported by Kamin and Lang (23) —our research offers novel evidence that Internet use among older adults in China is still less common. But, it is in line with previous studies that suggest that most Chinese older adults lack the digital literacy required to access the Internet, a novelty that appeared when they entered middle or old age (15). Despite many scholars expecting older adults to use the Internet to communicate with others and in turn obtain social support (29), our study identified reading the news as the most common activity for participants (9.0%), rather than chatting with others (6.9%). This echoes Western research showing older adults tend to use the Internet to access information, including news and health-related information (32).

Consistent with the literature (5, 7), our study confirms Chinese older adults who were socially isolated were also vulnerable to higher risks of cognitive impairment and depression. The relationship between social isolation and cognitive function in later life has not been adequately explored, especially in non-Western societies (48). Although quite a few studies conducted in China have identified the negative effects of social isolation on late-in-life depression, most constructed social isolation with a single measure (e.g., marital or cohabiting status) or multiple measures (e.g., family size, living with a spouse, frequency of contact with children, rural residence, participation of social activities) (49). Our findings advanced prior studies by adopting a reliable and valid instrument: the LSNS-6, which has been indicated as a good tool to screen for social isolation among older, community-dwelling Chinese adults (38). Furthermore, the results demonstrate isolation (e.g., infrequent contact with others) in both family and friendship ties was significantly associated with more depressive symptoms as well as cognitive impairment among Chinese older adults.

Our results shed light on the equivocal findings of the extant literature on the effects of Internet use (12, 13). They indicate Internet use was negatively associated with depression while it was positively related to cognitive function. Many scholars tend to explain the negative association between Internet use and depression by theorizing users are seeking emotional support or increased communication (17, 30). Our research demonstrates the Internet's pivotal role for Chinese older adults in providing information-based online activities. That is, among the study participants who reported often using the Internet, 86% did so to find information while 66% sought to chat with others. Thus, it is plausible that because the Internet in contemporary China has been widely used in public service and daily life, the Internet enables older users to access information themselves, which in turn, has positive effects on their psychological well-being. Connecting this result to the stress-coping framework, our findings underscore the significance of access to information as coping resources in relation to lowering depression levels among Chinese older adults. Western research has also confirmed informational support could allow older adults to accumulate the resources necessary to cope with common daily stressors and mitigate depressive symptoms (50).

Also in line with previous Western studies (22, 23), older adults in our sample who often used the Internet had a better cognitive performance. One possible explanation may be that as Kamin and Lang (23) suggested, older adults might obtain the cognitive benefits of online activities through handling technological tasks and challenges. Moreover, when using the Internet contributes to more cognitively stimulating environments, it can be a source of cognitive plasticity for older people (51). However, given the cross-sectional nature of our investigation, another possible explanation was suggested by Czaja et al. (52); they found cognition function would influence the use and adoption of digital technology in late life. In another word, keeping up with Internet developments and learning to use a new device were easier for older adults who were less cognitively impaired; thus, they are more likely to engage in more online activities.

Furthermore, our study went beyond existing knowledge and further indicates the path between social isolation and depression/cognitive function was moderated by older adults' Internet use. Those with lower Internet use exhibited a greater association between social isolation and worse cognitive function, compared to those using the Internet more. Similarly, older adults with lower Internet use reported a stronger association between social isolation and depression. One speculation is that as Elliot et al. (14) pointed out, the Internet use may have a moderating effect because it acts as a coping mechanism in response to late-life health challenges. Thus, Internet use may provide socially isolated older adults with greater opportunities for social support. Such a moderating effect of Internet use in gerontology has been examined by limited studies. Thus, our study advanced the gerontology literature and provided supporting evidence for the protective effects of Internet use among Chinese older adults against a specific stressor of being socially isolated. The current study further conducted the gender-specific analyses to examine whether the moderating effects of Internet use differ among females and males. The results show the moderating effect of Internet use was significant for both males and females. Additionally, among those who use the Internet more, the depression level of socially isolated male participants was much lower than for females. One possible explanation may be Internet use might buffer the effects of social isolation on late-in-life depression for males, compared with females. However, this finding echoes the suggestion of Hunsaker and Hargittai (20). Future research on a more nuanced look at the relationship between gender and Internet use among older adults is needed.

Certain limitations should be considered when interpreting our findings. First, the cross-sectional nature of the data could preclude unequivocal conclusions regarding the causal relationships between various variables (e.g., social isolation, Internet use) and late-in-life depression/cognitive function. Future studies should employ a longitudinal design to further establish the validity of temporal relationships and rule out potential bias. Second, the limitation of secondary data prohibited us from including more online activities (e.g., searching for health-related information, online dating), which have been explored in Western studies. Further investigation with more items to fully capture the diversity of Internet use by older adults is needed to replicate our results and to explain the moderating role of Internet use. Third, as described previously, our study conducted gender-specific analyses and found the moderating effect of Internet use might be more protective for males, but the gender differences in this topic are still not fully explained. Hence, further attention is warranted on aging populations' heterogeneity in Internet use, especially gender differences.

Despite these limitations, our study offers significant, practical implications. Overall, our findings suggest that Internet use could buffer the negative effects of social isolation on increasing depression and cognitive impairment among older adults. It highlights the importance of improving Internet accessibility, digital literacy, and positive attitudes of Chinese older adults, especially those who are socially isolated. First, due to the late popularization of the Internet, a digital divide exists among Chinese older adults whereby older adults with higher socioeconomic status, including higher level of education or income, or an urban residence (53) are more likely to access the Internet. Given the protective role of Internet use seen in our findings, it is urgent to remove barriers at the macro level through increasing the coverage of infrastructure in rural areas and providing free Internet for older adults. Also, the design of computers, the Internet, and mobile communication devices should consider older adults' characteristics, including cognitive abilities, declining visual and auditory abilities, and use habits, to design more age-friendly products. Second, because face-to-face social support services (peer support groups) might be less applicable during the COVID-19 pandemic, the Internet and social media apps could serve as an alternative for coping with social isolation or loneliness. Hence, to prevent negative experiences due to technical difficulties, frontline practitioners should engage older adults in technical training programs to help them improve their digital literacy and build their capacity to use the Internet. Finally, our study implies that older adults who are more socially isolated might benefit more from using the Internet. Considering the high proportion of empty nests among Chinese older adults, a targeted technical training service on a nationwide scale for older adults (particularly those with narrow social networks and “empty-nest elders”) may be a necessary step to prevent or reduce the adverse impacts of social isolation.

## Conclusion

In conclusion, the current study identified that Internet use among older adults in China is still less common, with 11.4% often using the Internet to engage in at least one activity. And reading the news was the top usage for Chinese older adults, instead of chatting with others. Our results shed light on the equivocal findings of the effects of Internet use and indicated Internet use was negatively associated with depression while it was positively related to cognitive function. We further expanded our understanding and indicated that Internet use might buffer the negative effects of social isolation on increasing depression/cognitive impairment among older adults. Recommendations for service practice and future research are discussed.

## Data Availability Statement

The data analyzed in this study is subject to the following licenses/restrictions: We need to seek consent of the CLASS research team to disclose the data to the journal if our paper is accepted. Requests to access these datasets should be directed to prof. Zhai Zhenwu, zhaizw@ruc.edu.cn.

## Author Contributions

YL conducted the first statistical analysis. All authors discussed paper structure and contributed to different part of the literature and composed the first draft together.

## Funding

The study was supported by China Social Academy Research Fund (Grant no. 21BSH130) and will support the potential fee for publication.

## Conflict of Interest

The authors declare that the research was conducted in the absence of any commercial or financial relationships that could be construed as a potential conflict of interest.

## Publisher's Note

All claims expressed in this article are solely those of the authors and do not necessarily represent those of their affiliated organizations, or those of the publisher, the editors and the reviewers. Any product that may be evaluated in this article, or claim that may be made by its manufacturer, is not guaranteed or endorsed by the publisher.
